# Association between carbohydrate consumption and self-assessed severe headache or migraine among American adults

**DOI:** 10.1017/jns.2026.10081

**Published:** 2026-04-15

**Authors:** Deqi Zhai, Saichun Zhang, Zihua Gong, Yingyuan Liu, Wenting Zhai, Longteng Ma, Shuqing Wang, Xiaoxue Lin, Shuhua Zhang, Xiu Liu, Huijuan Yuan, Yahui Zhu, Ludan Chen, Huanxian Liu, Zhao Dong

**Affiliations:** 1 Department of Neurology, Chinese PLA General Hospital First Medical Center, China; 2 International Headache Center, Chinese PLA General Hospital First Medical Center, China; 3 Department of Endocrinology, Chinese PLA General Hospital First Medical Center, China; 4 Department of Neurology, Bethune International Peace Hospital, China; 5 Department of Neurology, Peking Union Medical College Hospital, Beijing, China; 6 Affiliated Hospital, Heze Medical College, China; 7 Chinese PLA Medical School, China; 8 Department of Neurology, Beijing Electric Power Hospital, Beijing, China; 9 Department of Neurology, The First Affiliated Hospital of Chongqing Medical University, Chongqing, China; 10 Department of Neurology, Xinqiao Hospital and The Second Affiliated Hospital, Army Medical University, Chongqing, China; 11 Department of Neurology, Beijing Tiantan Hospital, Capital Medical University, Beijing, China; 12 Department of Neurology, Beijing Shijitan Hospital, Capital Medical University, Beijing, China; 13 School of Medicine, Nankai University, China

**Keywords:** Carbohydrate consumption, Cross-sectional study, Headache, Migraine

## Abstract

Prior research suggests that low-carbohydrate diets may reduce the frequency of headache attacks in individuals with migraine. However, the association between dietary carbohydrate intake and migraine in adults remains unclear. Given migraine’s significant public health burden and the modifiable nature of diet, understanding this relationship is vital for prevention. This study therefore investigated whether carbohydrate intake is associated with severe headache or migraine in a nationally representative sample of US adults. Using National Health and Nutrition Examination Survey (NHANES) data (1999–2004), this study examined the association between dietary carbohydrate intake and severe headache or migraine in adults aged over 20. Multivariable logistic regression was used, adjusting for demographics, socioeconomic status, lifestyle factors, and comorbidities. The study surveyed 10,413 participants, with 2062 reporting severe headache or migraine. Analysis of carbohydrate energy percentage revealed: compared to Q1 (≤42.7%), odds ratios (ORs) for severe headache or migraine were 1.04 for Q2 (42.7% to ≤50.5%, *P* = 0.642), 1.13 for Q3 (50.5% to ≤58.0%, *P* = 0.176), and 1.32 for Q4 (>58.0%, *P* = 0.008). A non-linear association was found between dietary carbohydrate intake and severe headache or migraine among U.S. adults (*P* for non-linearity = 0.002). The group with carbohydrate intake ≥51.1% of total energy had an OR of 1.22 (95% CI: 1.09–1.38, *P* = 0.002) compared to those below this level. The data suggest a significant association, with an important inflection point occurring at approximately 51.1%. This research uncovered a non-linear link between carbohydrate intake from diet and the chance of suffering from severe headache or migraine among American adults.

## Graphical abstract

**Figure:**
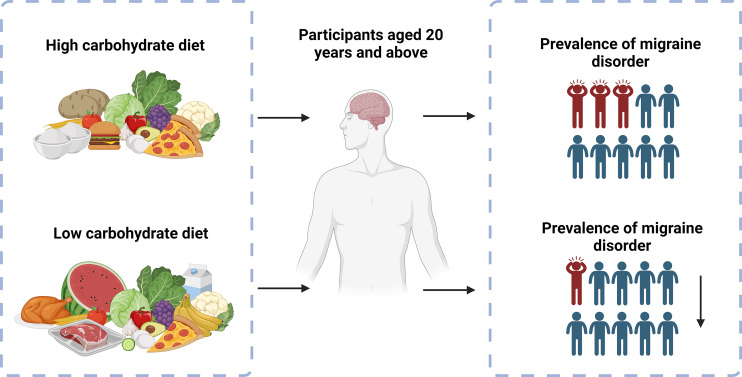

## Introduction

Globally, migraine ranks second among causes of disability^(1)^ and significantly contributes to economic burdens on society^(2)^. This underscores the urgent need for diverse programmes aimed at preventing and treating migraine disease. Recent reviews have highlighted a association between migraine and diet,^(3,4)^ suggesting that varying dietary patterns or interventions can influence headache attacks in persons with migraine disease.^(5,6)^

Among the six essential nutrients, carbohydrates – serving as the primary energy source for the human body – have garnered particular attention in migraine research. Previous studies have demonstrated that fasting states or excessive carbohydrate intake may directly trigger migraine attacks, while clinical evidence from low-carbohydrate diets (e.g., healthy diets, low-glycaemic index diets, and ketogenic diets) suggests a potential reduction in headache frequency.^(7)^ For instance, the ketogenic diet, which drastically reduces carbohydrate intake and increases fat proportion, may alleviate migraine symptoms by inhibiting the release of calcitonin gene-related peptide (CGRP) or modulating central nervous system excitability.^(8,9)^ This targeted intervention effect – specifically adjusting carbohydrate ratios without altering other nutrients – further underscores the unique role of carbohydrates in migraine management.

Furthermore, a global dietary trend has led to a significant increase in the consumption of refined carbohydrates (e.g., added sugars and processed foods), which is closely linked to the epidemic of metabolic disorders such as obesity and diabetes, conditions characterized by chronic inflammatory states,^(10,11)^ and the type and quantity of carbohydrate intake may serve as a critical bridge connecting these comorbidities to the pathogenesis of migraine. Thus, focusing on carbohydrates rather than other nutrients aligns more closely with the potential impact of modern dietary patterns on the burden of migraine.

Although existing studies suggest potential benefits of carbohydrate modulation in migraine (e.g., clinical applications of the ketogenic diet),^(8,9)^ systematic data on the specific associations between carbohydrate intake and headache in the U.S. adult population remain insufficient. Epidemiological evidence indicates that high-carbohydrate diets are significantly associated with elevated systemic inflammatory markers,^(12)^ whereas complete suppression of carbohydrate metabolism may markedly reduce inflammatory responses.^(13)^ Clinical trials further demonstrate that such interventions achieve relatively high migraine relief rates,^(7–9)^ suggesting a possible dose-response relationship between carbohydrate intake and migraine attack frequency.

The U.S. dietary pattern is characterized by high consumption of processed foods and high-glycaemic index carbohydrates,^(14)^ making it an ideal population for investigating the carbohydrate-migraine relationship. Leveraging large-scale epidemiological data such as the National Health and Nutrition Examination Survey (NHANES), this study aims to validate a dose-response relationship between carbohydrate intake and migraine attack frequency, addressing current gaps in regional and population-specific research. We hypothesize that increased carbohydrate intake is positively correlated with the frequency of migraine attacks.

## Methods

### Origin of the data

Led by the Centers for Disease Control and Prevention in the United States, NHANES is a cross-sectional epidemiological study that applies a layered multi-phase probability sampling method.^(15)^ The sampling process involves multiple stages: primary sampling units (counties or contiguous county groups) are first selected, stratified by geographic and socioeconomic criteria. Segments – such as census blocks or household clusters – are then chosen within these units, followed by random household selection. Eligible individuals within households are subsequently invited to participate. To enhance generalizability to the U.S. population, specific subgroups (e.g., racial/ethnic minorities, older adults, and low-income populations) are oversampled. This complex survey design ensures representativeness but requires the application of sampling weights in all analyses to account for differential selection probabilities, non-response, and oversampling. Started in 1999, NHANES runs on a two-year cycle, amassing comprehensive information about the health and nutrition of individuals within the U.S. demographic. This includes demographic details, physical examination outcomes, laboratory results, and dietary habits.^(16)^ An ethics review committee has granted approval for the data collection of NHANES, which is managed by the National Center for Health Statistics.^(17)^ As of September 2024, data analysis for the current research was conducted. Secondary data analyses do not require additional institutional review board approval, due to written informed consent is mandatory for all NHANES participants.

**Figure 1.**
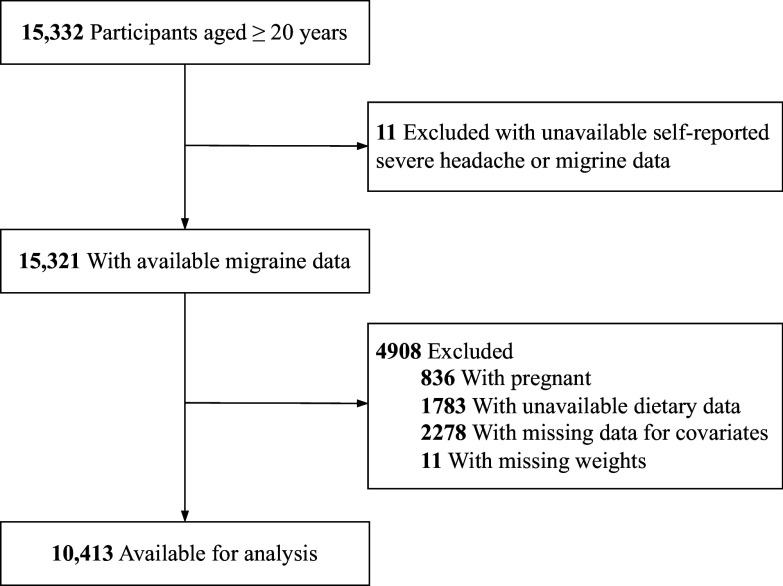
Inclusion and exclusion process of the study was based on the 1999–2004 National Health and Nutrition Examination Survey.

NHANES data can be accessed by the public through their official website (https://www.cdc.gov/nchs/nhanes/) (accessed on 4 September 2024). We based our study on adult participant data from the NHANES cycles spanning 1999 to 2004, selected for their inclusion of severe headache or migraine data within the miscellaneous pain questionnaire. This study focused on participants aged 20 years and above who had completed the interview process. To ensure the reliability of key outcome variables (severe headache or migraine status) and confounding factors (dietary carbohydrate consumption and baseline health covariates), individuals meeting any of the following exclusion criteria were excluded from the analysis: first, those with unavailable self-reported severe headache or migraine data (*n* = 11), as these primary outcome measures were essential for the study’s objectives; second, pregnant participants (*n* = 836), because oestrogen secretion levels during pregnancy could alter participants’ baseline headache status;^(18)^ third, individuals lacking dietary data (including carbohydrate consumption, *n* = 1783), since carbohydrate intake in dietary records was a critical variable under investigation in this study; fourth, participants with missing data for important baseline covariates (*n* = 2278), where “important covariates” specifically included age, sex, body mass index (BMI), blood pressure, and comorbid conditions identified in prior headache epidemiological studies as strongly associated with migraine prevalence or chronic headache outcomes, necessitating their inclusion to adjust potential confounding effects in causal inference;^(19,20)^ and finally, those with missing weight measurements (*n* = 11), as weight data were required for BMI calculation, a key covariate in analysing the relationship between headaches and dietary factors. The final analytical sample comprised 10,413 individuals, as detailed in Figure 1.

### Severe headache or migraine and carbohydrate consumption

This study identified individuals with a history of severe headache or migraine by analysing their responses to a particular inquiry located in the miscellaneous pain section of the NHANES survey: “Have you had a severe headache or migraine in the past three months?”^(19,21,22)^ In dietary evaluations, NHANES relies on the Automated Multiple Pass Method during the execution of 24-hour food recall surveys. This system, with computer assistance, thoroughly records the food and beverage intake of participants for the preceding 24-hour period, starting at midnight and ending at midnight.

Carbohydrate intake density in the diet was assessed using data from the USDA’s Food and Nutrient Database for Dietary Studies. The calculation of carbohydrate energy percentage, defined as the proportion of energy derived from carbohydrates (expressed as % of kcal), is based on the assumption that carbohydrates provide 4 kcal/g. This percentage is calculated using the formula: carbohydrate energy percentage (% of kcal) = carbohydrates intake (g/day) × 4 (kcal/g)/energy intake (kcal/day).^(23)^

### Covariates

A range of possible covariates were considered in our study, informed by existing research literature. These encompassed age, BMI, gender, spousal status, racial/ethnic background, level of education, dietary intake, household financial status, smoking behaviour, alcohol consumption, physical activity, diabetes, cardiovascular condition (confirmed by a doctor), and metabolic syndrome.^(19,20,22,24–26)^ The poverty income ratio (PIR) was used as the basis for classifying family income into low (PIR ≤ 1.3), medium (PIR 1.3–3.5), and high tiers (PIR > 3.5), according to a US government publication.^(27)^ Following the guidelines from previous research, participants were categorized as never, former, or current smokers.^(25)^ Alcohol consumption was similarly classified into never, former, or current drinker. Engaging in moderate to vigorous exercise for over 10 minutes during the last 30 days was considered physical activity; participants who did not meet this criterion were labelled as physically inactive. A 24-hour dietary recall interview, conducted at the mobile examination centre, provided detailed information on the participants’ total energy, protein, total fat, and dietary fibre intake. The diagnosis of diabetes was confirmed if any of the following criteria were met: a doctor’s diagnosis, Plasma glucose levels of ≥11.1 mmol/L in a 2-hour oral glucose challenge, ≥11.1 mmol/L in a random test, ≥7.0 mmol/L when fasting, a glycosylated haemoglobin (HbA1c) greater than 6.5%,^(28)^ or being treated with insulin or diabetes medication. Participants were classified as having diabetes if they met at least one of the above criteria. To resolve potential conflicts in classification (e.g., a participant on medication with a normal HbA1c), a pre-defined hierarchy was used: treatment with glucose-lowering medication was given the highest priority, followed by a self-reported doctor’s diagnosis, and then any abnormal biochemical value. This hierarchy ensures that individuals receiving treatment for diabetes are appropriately classified regardless of their current biochemical status. Participants identified solely by elevated biochemical markers, without a prior diagnosis or treatment, were defined as having undiagnosed diabetes. Individuals with missing data for all diabetes-related variables were excluded from the analysis. In this study, metabolic syndrome was described based on the 2005 Adult Treatment Panel III criteria.^(29)^

### Statistical analyses

This research involved a reanalysis of an open-access dataset, following the analytical guidelines established by NHANES.^(30)^ Our analysis meticulously accounted for the complex sampling design and associated sampling weights. We utilized dietary weights in our weighted analysis. Specifically, NHANES data from the 1999–2000 and 2001–2002 cycles were analysed with the four-year dietary weighting system (WTDR4YR), whereas the first-day dietary weighting system (WTDRD1) was used for the 2003–2004 data. The determination of sample weight values over the 1999–2004 timeframe was based on the following scheme: for the years 1999–2002, weights were calculated as 2/3 × WTDR4YR, and for the subsequent years, as 1/3 × WTDRD1. This weighting scheme is consistent with the NHANES analytical guidelines for combining multiple survey cycles. The periods 1999–2000 and 2001–2002 constitute 4 years of data and were therefore assigned a four-year dietary weight (WTDR4YR). The 2003–2004 period constitutes 2 years of data and was assigned a first-day dietary weight (WTDRD1). To create a composite weight that accurately represents the U.S. population over the entire 6-year study period (1999–2004), the original weights were proportionally scaled. The weight for the 1999–2002 data was multiplied by 2/3 (4 years/6 years), and the weight for the 2003–2004 data was multiplied by 1/3 (2 years/6 years). This adjustment ensures that each sub-period contributes to the analysis in proportion to its sample size and time span, preventing over- or under-representation of any single cycle and providing unbiased national estimates for our study window.

In this analysis, means and standard errors were used to describe continuous variables, while unweighted counts and weighted proportions were applied to represent categorical variables. We utilized linear regression models for numeric variables and χ^2^ tests for categorical data to assess variations between groups. A regression model with multiple variables was employed to investigate the relationship, and the findings are presented as odds ratios (OR) with 95% confidence intervals (95% CI) for the link between carbohydrate consumption and severe headache or migraine prevalence.

We constructed three multivariable regression models using a sequential adjustment strategy to comprehensively assess the association of carbohydrate consumption with self-assessed severe headache or migraine, and to evaluate the potential influence of different confounding pathways. This approach allows for the examination of how the effect estimate changes upon the addition of groups of covariates that represent different types of confounders, while avoiding over-adjustment for potential mediators.^(31,32)^ Model 1 (Minimal adjustment): This baseline model adjusted for demographic and basic socioeconomic factors, including age, gender, racial/ethnic background, marital status, level of education, and household financial status, as well as NHANES survey cycles. These factors are considered fundamental upstream confounders in health disparities research.^(33)^ Model 2 (Model 1 + Lifestyle factors): This model further adjusted for modifiable behavioural and anthropometric risk factors, including smoking status, BMI, alcohol use, and physical activity. This step was taken to test whether the observed association was independent of these major lifestyle-related confounders. Model 3 (Model 2 + Clinical comorbidities): This fully adjusted model additionally included history of clinical conditions: diabetes, metabolic syndrome, and cardiovascular disease. These factors were added last because they are potential mediators on the causal pathway between the exposure and the outcome. Adjusting for them in the final model allows us to examine if the exposure exerts an effect beyond or independent of these advanced disease states.^(31,32)^

To investigate a possible non-linear association between the proportion of energy from carbohydrates and severe headache or migraine, we built a regression model adjusted for multiple variables, using a restricted cubic spline with three knots. After adjusting for covariates in Model 3, we used a two-piece logistic regression model with smoothing to evaluate the threshold relationship between dietary niacin intake and migraine, using the median percentage of carbohydrate intake in total energy as the reference point. The inflection points were identified using likelihood-ratio tests and the bootstrap resampling method.

Additionally, to assess the potential effect modification by key demographic and metabolic factors, we conducted stratified analyses based on a priori hypotheses. Sex was selected due to well-established differences in headache prevalence and pathophysiology linked to hormonal influences.^(34,35)^ Age was stratified at 50 years to approximate the menopausal transition in women and to explore age-related metabolic changes. Household financial status was chosen as a determinant of dietary quality and access to healthcare, which may confound or modify the diet-headache relationship.^(36)^ Body Mass Index (BMI) was included because obesity is a pro-inflammatory state and a known risk factor for headache chronification, which may alter susceptibility to dietary triggers.^(37,38)^ Specifically, we stratified by sex, age (20–50 years and ≥50 years), household financial status (low vs. middle or high), and BMI (<25 kg/m² and ≥25 kg/m²). We separately applied logistic regression models and likelihood ratio tests to formally assess statistical heterogeneity and interaction across these subgroups.

Statistical power calculations were not conducted a priori due to the sample size being solely determined by the available data. We used R software version 4.2.1, in conjunction with the R survey package version 4.1-1 and Free Statistics software version 1.7.1, to perform statistical analyses.^(39)^ Statistical significance was evaluated through a two-tailed *P*-value below 0.05.

## Results

### Study population

During the three NHANES cycles spanning 1999–2000, 2001–2002, and 2003–2004, 15,332 participants aged 20 years and above were enrolled. A total of 11 individuals were omitted from this study due to data gaps related to severe headache or migraine. Additionally, 836 women in pregnancy, 1783 individuals without dietary carbohydrate intake data, 2278 missing covariate data, and 11 lacking appropriate weighting data were also excluded from the study. As depicted in Figure 1, the analysis ultimately incorporated 10,413 participants from the NHANES cycles between 1999 and 2004.

### Baseline characteristics

Among the 10,413 subjects enrolled in this research, 2062 experienced severe headache or migraine, while 8351 didn’t report such conditions. As shown in Table 1, the baseline characteristics of these individuals are categorized by carbohydrate energy percentage quartiles. The average age was 46.6 ± 0.3 years, and 5114 (49.1%) of the participants were female.

**Table 1.** Characteristics of participants by quartiles of the carbohydrate energy percentage in the NHANES 1999–2004 cycles^a^

Characteristics	Total	Quartiles of carbohydrate intake, % of kcal				
		Q1(<42.7)	Q2(42.7 to 50.5)	Q3(50.5 to 58.0)	Q4(≥58.0)	*P*-value^b^
No.	10,413	2618(25.1)	2603(25.0)	2624(25.2)	2568(24.7)	
Age (years)	46.6(0.3)	46.8(0.4)	46.8(0.5)	47.0(0.5)	45.5(0.6)	0.1
BMI (kg/m^2^)	28.1(0.1)	28.8(0.2)	28.1(0.2)	27.9(0.2)	27.5(0.2)	<0.001
Sex						<0.001
Female	5114(49.1)	1105(43.5)	1264(50.1)	1331(52.4)	1414(57.3)	
Male	5299(50.9)	1513(56.5)	1339(49.9)	1293(47.6)	1154(42.7)	
Marital status						0.05
Living alone	3948(37.9)	1005(36.2)	938(33.6)	962(35.4)	1043(39.0)	
Married or living with a partner	6465(62.1)	1613(63.8)	1665(66.4)	1662(64.6)	1525(61.0)	
Race/ethnicity						<0.001
Non-Hispanic White	5480(52.6)	1441(77.5)	1436(76.3)	1376(73.5)	1227(69.9)	
Non-Hispanic Black	1975(19)	547(10.2)	473(9.8)	478(10.7)	477(11.0)	
Mexican American	2257(21.7)	485(5.7)	539(6.4)	596(7.9)	637(7.5)	
Others	701(6.7)	145(6.6)	155(7.5)	174(7.9)	227(11.6)	
Education level (years)						<0.001
<9	1536(14.8)	315(5.4)	335(4.9)	401(6.9)	485(8.7)	
9–12	4176(40.1)	1063(37.8)	1094(41.3)	1043(37.9)	976(37.9)	
>12	4701(45.1)	1240(56.9)	1174(53.8)	1180(55.2)	1107(53.4)	
Dietary intake						
Energy intake (kcal/d)	2200.5(14.0)	2311.2(31.7)	2288.5(30.4)	2191.0(26.8)	1994.5(25.8)	<0.001
Carbohydrate (g/d)	271.4(2.1)	205.7(3.2)	266.9(3.6)	296.6(3.5)	322.2(3.9)	<0.001
Protein (g/d)	82.1(0.6)	98.9(1.2)	88.5(1.3)	77.6(1.1)	61.4(0.9)	<0.001
Total fat (g/d)	82.7(0.6)	103.6(1.3)	91.9(1.3)	78.1(1.0)	54.6(1.0)	<0.001
Dietary fibre (g/d)	15.8(0.2)	13.6(0.3)	16.0(0.3)	16.6(0.3)	17.3(0.4)	<0.001
PIR						<0.001
≤1.3	2901(27.9)	652(17.5)	690(20.4)	731(22.1)	828(25.8)	
1.3–3.5	4051(38.9)	968(33.1)	1021(35.8)	1057(37.5)	1005(36.2)	
>3.5	3461(33.2)	998(49.3)	892(43.8)	836(40.4)	735(38.0)	
Smoking status						<0.001
Never	5217(50.1)	1099(42.4)	1271(48.4)	1410(53.4)	1437(54.4)	
Former	2848(27.4)	748(27.3)	768(28.1)	689(25.0)	643(22.6)	
Current	2348(22.5)	771(30.4)	564(23.5)	525(21.6)	488(23.0)	
Alcohol consumption						<0.001
Never	1493(14.3)	240(7.2)	339(11.6)	409(13.9)	505(19.1)	
Former	2162(20.8)	437(13.6)	510(15.9)	583(19.5)	632(19.6)	
Current	6758(64.9)	1941(79.2)	1754(72.5)	1632(66.6)	1431(61.4)	
Physical activity						0.3
Active	5951(57.1)	1527(64.0)	1517(66.0)	1514(66.3)	1393(63.4)	
Inactive	4462(42.9)	1091(36.0)	1086(34.0)	1110(33.7)	1175(36.6)	
Diabetes	1423(13.7)	417(12.2)	361(10.2)	315(7.8)	330(8.4)	
Cardiovascular disease	1241(11.9)	278(8.6)	285(9.1)	347(11.1)	331(9.8)	
Metabolic syndrome	2977(28.6)	742(27.1)	751(26.4)	751(26.5)	733(25.7)	
Severe headache or migraine	2062(19.8)	459(18.8)	477(20.4)	536(22.3)	590(26.1)	

Abbreviation: PIR: family poverty income ratio; BMI: body mass index.

a Data for categorical variables are shown as unweighted counts (weighted percentages), and for continuous variables, as mean values (standard error).

b *P*-values were calculated from the one-way ANOVA or K-W test for Numeric variables and χ^2^ test for categorical variables.

Participants with higher carbohydrate energy percentage were predominantly female, more often of Mexican American or other races, and tended to consume fewer calories, proteins, and fats, but more fibre. They also generally had lower education levels, lower family incomes, and were more likely to have never smoked or consumed alcohol.

### Association between carbohydrate consumption and severe headache or migraine

Our analysis of the association between the percentage of energy from carbohydrates and severe headache or migraine was conducted using three progressively adjusted models (Table 2). The association remained significant and showed an increasing trend across all models. Model 1 (Adjusted for demographic and socioeconomic factors): OR = 1.53 (95% CI: 1.28–1.82, *P* < 0.001). Model 2 (Further adjusted for lifestyle factors): OR decreased to 1.28 (95% CI: 1.06–1.56, *P* = 0.014). This suggests that after accounting for demographic factors, the inclusion of lifestyle factors such as smoking status and BMI had a weakening effect on the association. Model 3 (Fully adjusted model, including medical conditions): OR increased again to 1.32 (95% CI: 1.08–1.61, *P* = 0.009). This indicates that, after controlling for demographic and lifestyle factors, the inclusion of medical conditions such as diabetes and metabolic syndrome enhanced the association, and the relationship remained statistically significant in the fully adjusted model. This trajectory clearly illustrates the independent influence of different categories of confounders: lifestyle factors tend to attenuate the association, while specific medical conditions tend to strengthen it. This highlights the complex, multifactorial nature of the relationship between carbohydrate intake and severe headache or migraine.

**Table 2.** Association between dietary carbohydrate intake and severe headache or migraine among adult participants in the NHANES 1999–2004 cycles

Quartiles	OR (95% CI)								
	No.	Crude	*P*-value	Model 1^a^	*P*-value	Model 2^b^	*P*-value	Model 3^c^	*P*-value
Carbohydrate intake, % of kcal	10,413	1.16(1.10,1.22)	<0.001	1.10(1.04,1.17)	0.002	1.12(1.05,1.19)	0.001	1.11(1.05,1.18)	0.002
Q1(<42.7)	2618	1(Ref)		1(Ref)		1(Ref)		1(Ref)	
Q2(42.7 to 50.5)	2603	1.11(0.93,1.32)	0.230	1.01(0.84,1.22)	0.894	1.05(0.87,1.26)	0.612	1.04(0.86,1.26)	0.642
Q3(50.5 to 58.0)	2624	1.24(1.05,1.46)	0.013	1.11(0.93,1.33)	0.238	1.15(0.96,1.39)	0.127	1.13(0.94,1.37)	0.176
Q4(≥58.0)	2568	1.53(1.28,1.82)	<0.001	1.28(1.06,1.56)	0.014	1.33(1.10,1.62)	0.005	1.32(1.08,1.61)	0.008
Trend test			<0.001		0.014		0.006		0.009

a Model 1 was adjusted for age, sex, race/ethnicity, education level, marital status, family income, and NHANES cycle.

b Model 2 was adjusted for model 1 + smoking status, BMI, alcohol consumption, and physical activity.

c Model 3 was adjusted for model 2 + diabetes, metabolic syndrome, and cardiovascular disease.

**Figure 2.**
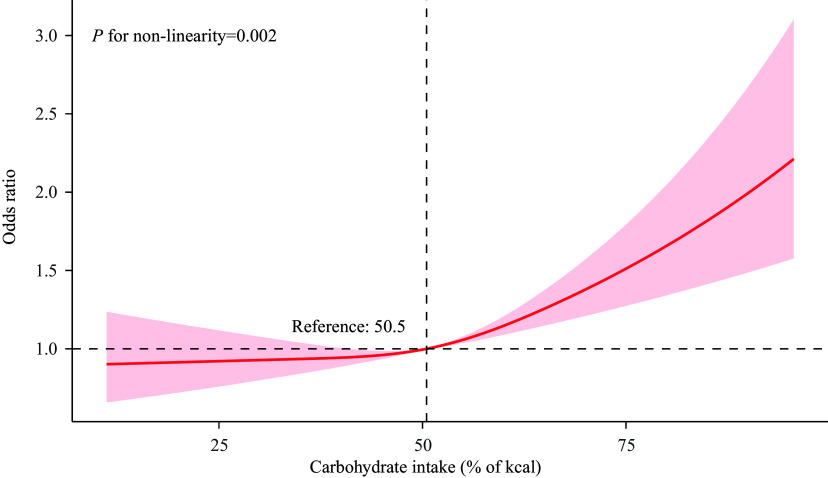
The dose–response relationship between carbohydrate energy percentage and severe headache or migraine. Solid and dashed lines indicate the predicted value and 95% CI. The restricted cubic spline model was adjusted for age, sex, race/ethnicity, education level, marital status, family income, NHANES cycle, smoking status, BMI, alcohol consumption, physical activity, diabetes, metabolic syndrome, and cardiovascular disease. NHANES, National Health and Nutrition Examination Survey.

Figure 2 illustrates the non-linear association between carbohydrate energy percentage and the risk of severe headache or migraine (*P* for non-linearity = 0.02). The relationship, modelled using restricted cubic splines, reveals a J-shaped curve, indicating that the risk is relatively stable at lower carbohydrate energy percentages but increases significantly beyond a threshold of approximately 50.5% of total energy intake. The OR for headache/migraine risk is low with carbohydrate energy contribution below 50.5%.

**Figure 3.**
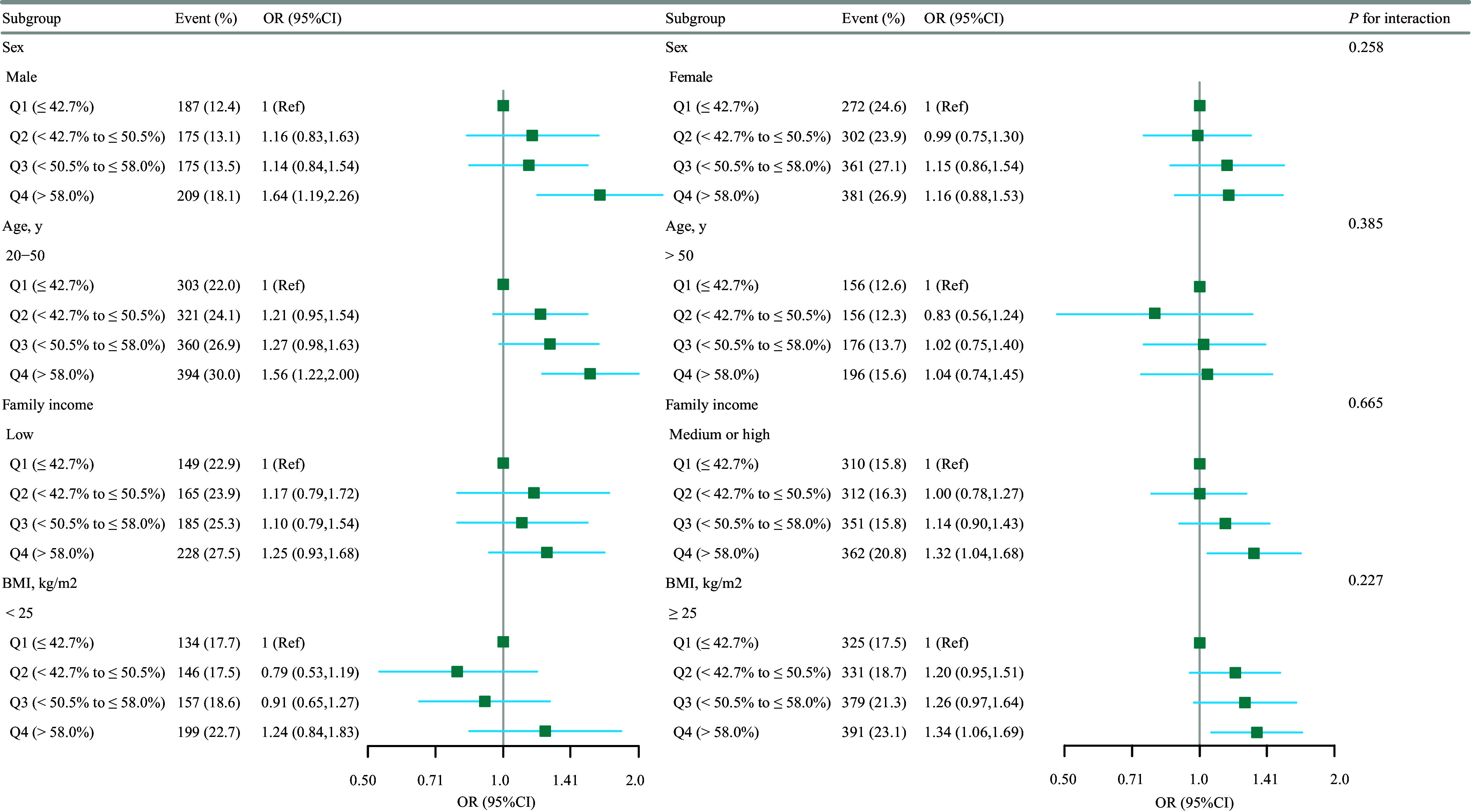
Association between the carbohydrate energy percentage and severe headache or migraine according to the general characteristics. Except for the stratification factor itself, the stratifications were adjusted for all variables (age, sex, race/ethnicity, education level, marital status, family income, NHANES cycle, smoking status, BMI, alcohol consumption, physical activity, diabetes, metabolic syndrome, and cardiovascular disease). BMI, body mass index; NHANES, National Health and Nutrition Examination Survey.

In the piecewise regression model (Table 3), the results showed that among individuals with a percentage of carbohydrate intake in total energy ≥51.1%, the adjusted OR for experiencing severe headache or migraine was 1.22 (95% confidence interval 1.09–1.38; *P* = 0.002). For those with a percentage of carbohydrate intake in total energy <51.1%, no significant association was observed.

**Table 3.** Association between the carbohydrate energy percentage and severe headache or migraine using two-piecewise regression models

Carbohydrate Intake, % of kcal	OR (95% CI)							
	Crude model	*P*-value	Model 1^a^	*P*-value	Model 2^b^	*P*-value	Model 3^c^	*P*-value
<51.1	1.09(0.95,1.24)	0.214	1.02(0.90,1.16)	0.746	1.04(0.91,1.19)	0.534	1.04(0.90,1.19)	0.596
≥51.1	1.28(1.13,1.44)	<0.001	1.22(1.08,1.38)	0.002	1.22(1.09,1.37)	0.002	1.22(1.09,1.38)	0.002

a Model 1 was adjusted for age, sex, race/ethnicity, education level, marital status, family income, and NHANES cycle.

b Model 2 was adjusted for model 1 + smoking status, BMI, alcohol consumption, and physical activity.

c Model 3 was adjusted for model 2 + diabetes, metabolic syndrome, and cardiovascular disease.

### Subgroup analyses

Our subgroup analysis aimed to assess the heterogeneity of the association between the percentage of energy from carbohydrates and severe headache or migraine (Figure 3). In this study, statistical significance was defined as a *P*-value <0.05. After stratifying the data by gender, age, BMI, and family income, we found:

Gender: In the male subgroup, there was a statistically significant positive association between the percentage of energy from carbohydrates and severe headache or migraine (OR = 1.64, 95% CI 1.19–2.26). However, this association did not reach statistical significance in the female subgroup (OR = 1.16, 95% CI 0.83–1.63). The *P*-value for the test of interaction between subgroups was 0.258, indicating that the difference in effects between gender subgroups was not statistically significant.

Age: A significant positive association was observed in the participant subgroup aged 20 to 50 years (OR = 1.56, 95% CI 1.22–2.00). In contrast, the association was not significant in participants older than 50 years (OR = 1.04, 95% CI 0.74–1.45). The *P*-value for the test of interaction between subgroups was 0.385, suggesting that the difference in effects by age stratification was not statistically significant.

BMI: For participants with a BMI ≥ 25 kg/m², we observed a significant positive association (OR = 1.34, 95% CI 1.06–1.69). For participants with a BMI < 25 kg/m², the association was not significant (OR = 0.79, 95% CI 0.53–1.19). The *P*-value for the test of interaction between subgroups was 0.50, indicating that the difference in effects by BMI stratification was not statistically significant.

Family Income: A significant positive association was observed in participants with medium or high family income (OR = 1.32, 95% CI 1.04–1.68). In the low-income participant subgroup, the association was not significant (OR = 1.10, 95% CI 0.79–1.54). The *P*-value for the test of interaction between subgroups was 0.227, indicating that the difference in effects by family income stratification was not statistically significant.

In summary, although significant risks associated with the percentage of energy from carbohydrates were observed in specific subgroups (e.g., males, aged 20–50, BMI ≥ 25 kg/m², medium/high-income groups), the tests for interaction between subgroups did not reach statistical significance (e.g., gender *P* = 0.258, age *P* = 0.385, etc.). Therefore, we cannot conclude that there is a statistically significant interaction between subgroups. This finding suggests that the percentage of energy from carbohydrates may have a specific impact on certain populations, but the heterogeneity of its effects has not been confirmed by the current statistical tests.

## Discussion

A non-linear correlation between carbohydrate energy percentage and the adult prevalence of severe headache or migraine is demonstrated in this U.S.-based national cross-sectional study. The robustness of this association is further affirmed through subgroup analyses.

Previous research has explored the link between dietary carbohydrate intake and migraine with mixed results. On one hand, several studies have suggested a potential association. For instance, one study indicated a relationship between the frequency of migraine attacks in children and their consumption of high-fat or high-sugar foods and beverages.^(40)^ Furthermore, some interventional studies have observed that dietary patterns with lower carbohydrate intake, such as the ketogenic diet, low-glycaemic diet, and the Healthy Eating Plate (HEP), may ameliorate migraine symptoms.^(4,6,41–44)^ The proposed mechanisms often involve blood glucose fluctuations; prior research suggests that consuming carbohydrates in fasting states or in large quantities can lead to reactive hypoglycaemia or hyperglycaemia, potentially triggering migraine attacks in susceptible individuals.^(45)^ Adding to this, a recent study suggested that high adherence to the Carbohydrate Quality Index, which emphasizes high-fibre, whole-grain carbohydrates, is associated with lower headache severity and duration.^(46)^ On the other hand, the evidence is not entirely consistent. Some systematic reviews have highlighted the limited quantity and moderate quality of studies supporting low-carbohydrate diets for migraine, calling for more robust evidence.^(47)^ Other large-scale observational studies have found no independent association between total carbohydrate or sugar intake and migraine risk after adjusting for potential confounders like obesity and depression,^(48)^ suggesting that the relationship may be more complex or indirect. Given this context of mixed findings, and considering that the specific correlation between carbohydrate consumption and migraine within the general U.S. population has not been extensively studied, our analysis aimed to contribute to this dialogue using nationally representative data. The current study, utilizing NHANES data, adjusted for a comprehensive set of potential confounders through multivariable regression analyses, thereby enhancing its relevance to the U.S. adult population. Even after controlling for these factors, our study indicates that a higher carbohydrate energy percentage is linked to a heightened risk of severe headache or migraine. Carbohydrate energy percentage and severe headache or migraine demonstrated a non-linear association, as elucidated by dose-response analyses. Specifically, carbohydrate energy percentages below 51.1% did not lead to an escalation in the risk of severe headache or migraine, suggesting that the risk increases only when carbohydrate energy percentage crosses this threshold. Moreover, the association remained consistent across different subgroups in our analyses. This threshold effect may help explain some of the heterogeneity in previous studies and underscores the importance of considering dosage and dietary patterns in future research.

The relationship between dietary carbohydrates and migraines is indeed an area of ongoing research, though not all findings are entirely conclusive. While several studies, including those we cited, suggest a potential link, some research has also pointed out the limitations of the existing evidence. For instance, a systematic review by Caminha demonstrated that a ketogenic diet reduced the frequency and severity of migraine attacks in patients, but it remains unclear whether the effects can be attributed solely to the treatment.^(49)^

Fasting and high carbohydrate intake may increase blood glucose fluctuations, potentially triggering migraine attacks.^(45)^ Research with male rats shows that insulin and glucagon can alter pain signal transmission in the trigeminal-cervical complex.^(47)^ This suggests a neurobiological link between migraine and fluctuations in blood glucose, as well as glucoregulatory hormones.^(48)^ These factors may contribute to the pathogenesis of migraine. Multiple animal studies suggest that neuroinflammation significantly impacts migraine pathophysiology.^(50–52)^ low-glycaemic diet has a lower proportion of carbohydrate intake. A clinical study examining the effects of a legume-rich, low-glycaemic diet on insulin resistance and inflammation markers found significant reductions in the levels of soluble tumour necrosis factor-alpha receptor II and C-reactive protein following adherence to low-glycaemic diet.^(53)^ This indicates that low-glycaemic diet may influence the inflammatory response, thereby alleviating migraine. Oxidative stress is closely associated with migraine.^(54–57)^ Moreover, a high-carbohydrate diet can promote oxidative stress.^(58–61)^ This indirectly suggests a close relationship between high-carbohydrate diets and the development of migraine. Furthermore, low-glycaemic diet shifts towards more resistant polysaccharides, resulting in a greater generation of short-chain fatty acids as the gut microbiota break them down, which could lower migraine frequency.^(62,63)^ Therefore, it’s hypothesized that high dietary carbohydrate intake may influence migraine through various mechanisms. However, longitudinal studies with rigorous designs are necessary to further explore this relationship.

In our subgroup analyses, we observed varying magnitudes of association between the percentage of energy from carbohydrates and the prevalence of severe headache or migraine across different demographic and socioeconomic strata. Specifically, statistically significant positive associations were identified in males, younger adults (aged 20–50 years), individuals with BMI ≥ 25 kg/m², and those with medium or high family income. By contrast, these associations were not significant in females, older adults, normal-weight individuals, or those with low family income. These divergent findings may reflect underlying biological and behavioural mechanisms. For instance, the stronger association observed in males could be related to sex differences in glucose metabolism and hormonal influences on pain pathways. Oestrogen in premenopausal women may offer some protective effect against carbohydrate-induced inflammation or neuronal excitability, which might attenuate the association in females.^(64)^ The age-related discrepancy may be attributed to metabolic flexibility and insulin sensitivity, which generally decline with age.^(65)^ Younger individuals may be more susceptible to dietary triggers such as high glycaemic load, which can promote neuroinflammation and migraine pathogenesis.^(66)^ The significant association in overweight or obese individuals (BMI ≥ 25 kg/m²) may be explained by the interplay among high carbohydrate intake, insulin resistance, and chronic low-grade inflammation – all of which are known contributors to headache disorders.^(67)^ Moreover, the association in medium- or high-income groups might reflect dietary patterns characterized by higher consumption of refined carbohydrates and processed foods, which are linked to increased risk of migraine.^(68)^ By contrast, low-income populations may have more constrained dietary choices or different competing risk factors, potentially diluting the observable effect of carbohydrate intake. Notably, however, tests for interaction across these subgroups did not reach statistical significance (e.g., P-interaction for sex = 0.258, age = 0.385, BMI = 0.50, income = 0.227). This indicates that while point estimates suggest variation, we cannot confidently conclude that the effect of carbohydrate intake differs meaningfully across subgroups based on the current data. The lack of significant interaction may be due to limited statistical power within strata or true homogeneity of the effect across populations. Therefore, while our findings suggest that certain groups may be more vulnerable to high dietary carbohydrate intake, the absence of significant interaction effects precludes definitive claims regarding heterogeneity. Future studies with larger samples and detailed dietary assessments are needed to explore these potential differences further and elucidate their biological underpinnings.

This study, however, is not without its limitations. Firstly, as it focuses on the U.S. population, additional research is necessary to determine if these results are applicable to other demographic groups. Secondly, while we have attempted to mitigate participation confounding effects through multivariable logistic regression models and subgroup analyses, these effects cannot be entirely discounted. Thirdly, although we adjusted for multiple covariates in our statistical analysis based on previous literature, the possibility that unmeasured or unrecorded confounding factors may affect our results cannot be excluded. Fourthly, the data on severe headaches or migraines in the NHANES database is derived from a single question: “Have you had a severe headache or migraine in the past three months?”. The scope of this question is limited, as it does not encompass the frequency, intensity, subtype (e.g., with or without aura), or genetic predisposition of the sufferers. This lack of phenotypic granularity presents a significant challenge. It restricts our capacity to explore whether dietary carbohydrates correlate specifically with distinct clinical characteristics of migraines (e.g., high-frequency vs. episodic). The use of this single metric again contributes to the non-differential misclassification discussed above, further biasing our effect estimates towards zero and limiting the clinical interpretability of our findings. Therefore, the primary impact of these methodological limitations is a reduction in the study’s power to detect a significant association and an underestimation of the true strength of the relationship between carbohydrate intake and migraine. Future prospective studies that employ validated headache questionnaires and International Classification of Headache Disorders criteria for participant phenotyping are essential to confirm and refine our findings, allowing for a more detailed investigation into how specific aspects of diet might influence specific migraine features. Fifthly, the use of a single 24-hour dietary recall to assess carbohydrate intake is a major limitation as it does not represent an individual’s long-term dietary patterns. About physical activity, our binary measure, while practical for a large-scale survey, cannot discern gradients of activity volume and does not reflect adherence to formal activity guidelines. We also suggest that future research with access to accelerometer-derived data (as later implemented in NHANES) would provide a more nuanced and accurate investigation into the relationship between physical activity levels and health outcomes. Sixthly, it is not feasible to determine a causal link between carbohydrate consumption and severe headache or migraine, due to the study’s cross-sectional design. Longitudinal studies are required to examine the potential causal relationship between carbohydrate consumption and the severity of headache or migraine. Finally, although we identified a significant non-linear association between the percentage of carbohydrate energy and the risk of headache or migraine using restricted cubic splines and piecewise regression models, we were unable to provide complete goodness-of-fit statistics (such as Akaike Information Criterion or Bayesian Information Criterion values) or direct comparative results between linear and non-linear models (e.g., χ² values and degrees of freedom for likelihood ratio tests). This omission was primarily due to the technical challenges associated with obtaining and interpreting these statistics in weighted regression analyses under NHANES’ complex multi-stage sampling design, as the results may be influenced by sampling weight adjustments. However, we partially mitigated this limitation by reporting a significant non-linearity test *P*-value (*P* for non-linearity = 0.02) and validating the findings using piecewise regression. Future studies should aim to refine model comparison methods under complex survey designs and report all relevant statistics to enhance the transparency and comparability of results.

## Conclusions

Our study identified a non-linear association between carbohydrate intake and severe headache or migraine, with an inflection point at 51.1% of energy. This threshold may hold practical significance for developing targeted dietary advice in the clinical management of these conditions. Future research should prioritize interventional trials to determine if modulating carbohydrate intake around this level can effectively mitigate headache risk.
